# Governing the soil: natural farming and bionationalism in India

**DOI:** 10.1007/s10460-022-10327-0

**Published:** 2022-07-08

**Authors:** Ian Carlos Fitzpatrick, Naomi Millner, Franklin Ginn

**Affiliations:** School of Geographical Sciences, University of Bristol, University Road, Bristol, BS8 1SS UK

**Keywords:** Soil, Alternative agriculture, Governmentality, Agroecology, Green revolution, Himachal Pradesh

## Abstract

This article examines India’s response to the global soil health crisis. A longstanding centre of agricultural production and innovation, India has recently launched an ambitious soil health programme. The country’s Soil Health Card (SHC) Scheme intervenes in farm-scale decisions about efficient fertiliser use, envisioning farmers as managers and soil as a substrate for production. India is also home to one of the world’s largest alternative agriculture movements: natural farming. This puts farmer expertise at the centre of soil fertility and attends to the wider ecological health of soils. Despite emerging as a mode of resistance to dominant agricultural systems, natural farming is now being delivered in increasingly bureaucratic ways by India’s state governments. This article offers Himachal Pradesh as a case study in how the soil is governed, drawing on 38 semi-structured interviews with scientists, agricultural officers, non-governmental organisation leaders, and activists. Rather than assess approaches to soil health according to their ecological bottom line, we examine the differing forms of knowledge, expertise and ‘truth’ in the SHC and Natural Farming approaches. Our analysis reveals discontinuities in how farmers are imagined, as well as continuities in how quasi-spiritual language combines in a bionationalist project, positing assumptions about the correct arrangement of life in nationalist terms. We point to a shift toward hybrid and pick-and-mix approaches to soil health, as farmers and their organisers are increasingly invested with the capacities to combine multiple options. We see a fracturing of expertise and the opening up of epistemic pluralism in responses to the soil fertility crisis.

## Introduction

In February 2015, Narendra Modi—India’s prime minister since 2014—launched an ambitious nationwide soil testing programme known as the Soil Health Card (SHC) scheme. The scheme provides information to farmers on the nutrient status of their soil and offers them fertiliser dose recommendations to improve soil health and productivity. The aim of the SHC is to address India’s soil crisis: overreliance on urea coupled with underuse of organic manure, has led to a “multi nutrient deficiency in India soil” (Ministry of Agriculture and Farmers Welfare 2016, p. 82). Nearly 30% of the country’s land area has been degraded through deforestation, over-cultivation, soil erosion and depletion of wetlands (Space Applications Centre 2016). In the first 6 years of the SHC (2015–2020), the government spent approximately 751 crore INR ($100.5 million) testing the soil of millions of farmers, expanding testing capacity by building new laboratories, and delivering hundreds of thousands of demonstrations. By any standard, this has been an enormous undertaking. There are signs that the SHC is having some impact. One study by the National Productivity Council found that the SHC recommendations had led directly to an 8–10% decline in the use of chemical fertilizer (Press Information Bureau 2018), although the fertilizer industry continues to grow by over 10% per year (Government of India 2020).

India’s SHC is one response to the global crisis in soil health. Over the past 20 years world agricultural production has increased threefold and the amount of irrigated land has doubled (Foley et al. 2011; Ellis et al. 2013). Heavy tilling, multiple harvests and abundant use of agrochemicals have increased yields at the expense of long-term sustainability. Global fertiliser use has increased by 500% (nitrogen by 800%) over the last 50 years (Foley et al. 2011). Consequently, a third of the planet’s land is severely degraded and fertile soil is being lost at the rate of 24 billion tonnes per year (UNEP/UNCCD 2016). The rift between ecological sustainability and agricultural production is growing: projections warn that land degradation and climate change together could reduce crop yields by up to 50% in some parts of the earth by 2050, forcing up to 700 million people to migrate (IPBES 2018).

At the same time, there is growing recognition that soil health is affected by more than just fertiliser use. Many historically marginalised cultivation and preparation techniques are available for managing soil health, including traditional knowledge practices such as mulching, leaving dung on soil and crop rotation. Since the 1980s, there has been huge growth in alternative farming systems in India and globally, including agroecology, organic farming, sustainable intensification, conservation agriculture, and minimum tillage or no-till systems. Key to an agroecological approach, for example, is the integration of ecological principles into the design and management of agroecosystems, with an emphasis on diversity (of crops) and complexity (where every component in the system plays multiple roles). Alternative agriculture is in part a response to the global soil crisis, but also to the extractivist approach of conventional agriculture and—in India especially—the legacies of the Green Revolution. In short, alternative agriculture is about more than economic or ecological bottom lines. It seeks to address the historic and ongoing alienation of small-scale farmers from their production and land caused by modern, industrial agriculture (Singh and Singh 2017).

The largest alternative farming movement in India, Zero Budget Natural Farming (ZBNF), began as a grassroots social movement. ZBNF aims to reduce dependence on external synthetic inputs and debt (zero budget) and farm on an agroecological basis (natural farming) (Bharucha et al. 2020, p. 5). In its most fervent versions, ZBNF invokes nationalist and nativist rhetoric, making explicit connections between farmer marginalisation, agri-business and the toxic legacies of twentieth-century agricultural modernisation. By combining some of the reparative principles and techniques found throughout the agroecology movement with a nativist agricultural narrative (Bhattacharya 2017, p. 550; Münster 2018, p. 750), ZBNF has developed a rapidly growing quasi-Hindu nationalist farmer-based social movement. Today, ZBNF has also evolved into a state-supported programme in several Indian states, including Himachal Pradesh. While this could be expected to bring farmer expertise and organic practice to the heart of subsidised practice, we assess how natural farming is reconfigured when it is instantiated as a state-level programme.

This paper analyses the contrasting approaches to governing the soil of state-promoted ZBNF and the SHC scheme. Our focus is on the similarities and differences in the rationales they employ to justify their approaches, the forms of knowledge on which they draw, and the ways they attempt to organise and discipline soil care. We are interested how the soil is made known and legible, and how it becomes worthy of intervention. We explore important continuities between the way this happens through ZBNF state programmes and the SHC programme, especially in the ways that both use bionationalism—understood as the use of biology and nativism to strengthen nationalist narratives—as a justifying rhetoric. Both also employ didactic learning techniques, whereby farmers are exhorted to behave in certain ways by outside interests (Stone 2016; Stone and Flachs 2017). Our analysis demonstrates the “fracturing” of expertise in soil management, beyond “unidirectional knowledge exchange” from scientific and institutional experts, into a domain where multiple epistemic and political claims are made as to how the soil should be governed (Krzywoszynska 2019, p. 161; Münster 2021).

We begin this paper by exploring the history of soil testing in India, linking the SHC scheme to the colonial legacy of systematically measuring and classifying the natural world. The subsequent section reviews the concept of governmentality as a tool for understanding the relationship between government and farmers’ soil management. After describing our methods and the case study of Himachal Pradesh, we then analyse the SHC and state-promoted approaches to ZBNF. In concluding, we reflect on the significance of these findings for soil health futures.

## A recent history of soil testing in India

India’s SHC scheme emerges from the legacies of colonial agricultural development. As Arnold (2000) notes, despite agriculture’s obvious role in subsistence and export economies, it was relatively overlooked by administrators before the 1890s. An influential report by the Royal Agricultural Society in 1893 warned that the government was “neglecting modern scientific approaches to agriculture, especially the contribution chemistry could make to improving India soils, manuring practices and crop yields” (cited in Arnold 2000, p. 151). Partly due to the 1876–1878 famine, a late nineteenth-century surge of interest in agricultural development occurred. Both central and provincial administrations created their own agriculture departments, although activities were initially limited to data collection and famine relief. In the late nineteenth century, agricultural and irrigation projects in the Punjab aimed to improve productivity, as well as to increase the state’s capacity to measure and control aspects of nature such as water and soil (Prakash 1999, p. 135). Further-reaching agricultural modernisation projects, such as the construction of the Lower Chenab Canal, required mapping and classification of land according to a range of variables such as population, rainfall, topography, and so on. Scott (1998, p. 2) famously describes the premodern state as partially blind: “it knew precious little about its subjects, their wealth, their landholdings and yields, their location, their very identity. It lacked anything like a detailed ‘map’ of its terrain and its people”. The extension of a “technological grid fashioned and administered by the state” (Prakash 1999, p. 170) relied on tools such as including the census (beginning 1872), measuring and surveying. Agricultural development went hand-in-hand with state development.

The first systematic soil survey was carried out in Madras in 1912, followed by the Punjab in 1919 (Nath 1941). At that time, an All-India survey was considered by the Royal Commission on Agriculture, but the high cost and existing knowledge of soil in important locations delayed its implementation. Post-independence, soil testing continued as a one among many government interventions aimed at technological innovation. One of the earliest documents aimed at creating uniformity of soil testing across the country was published in 1948 (Office of the High Commissioner for India 1948, p. 19). Sixteen soil testing laboratories followed, set up under an Indo-US operational agreement, between 1955 and 1956. Soil testing laboratories increased from 24 in 1958 to around 260 by 1977, including 52 mobile soil testing vans (Mutatkar 1980, p. 72). By the early 1990s there were more than 400 soil testing laboratories (Mahapatra 1996, p. 118), and over 500 by the early 2000s, including 118 mobile soil testing vans (Gupta 2004, p. 359), but the number has exploded in the last 20 years and there are now 3887 laboratories according to official government data (Government of India 2021a, b).

The focus of soil testing on a limited number of physical and biological components reduces soil’s complexity to a simplified representation (on Indian forestry, see Agrawal 2005). Drawing on such representations enabled the modern Indian state to cast itself as an instrument for public good and to reshape farming around the methods and data of modern soil science. Through the latter twentieth century, soil testing supported the Green Revolution. Green Revolution technologies, for which India was a centre of experimentation, sought to intensify agricultural production to tackle global hunger. While dramatically increasing production, new pest-resistant seeds quickly reduced the genetic diversity of crops. They also required ever higher inputs of chemical pesticides and fertilisers, as monocropping renders entire harvests more vulnerable to infestations and drains the mineral content of soils more rapidly (Patel 2013); fertiliser use in India more than quadrupled in a single decade, 1965–1975 (World Bank 2021). The state heavily subsidized electricity, fertiliser, machinery and other agricultural inputs, increasing farmers’ dependence on external inputs and production for urban markets.

Today India has over 1000 soil laboratories, with an annual capacity to test 10.7 million samples—a significant fraction of the country’s approximately 140 million individual landholdings (Swetha et al. 2020, p. 56). Soil testing laboratories are run by a combination of government agencies, universities and the private sector—including fertiliser manufacturers. Quantifying soil health in any accurate way is an extremely complex matter, with many chemical, physical and biological indicators needed. Additionally, standard soil testing does little to monitor the physical properties of soil or water holding capacity. Proposals have been made for even more technologically advanced methods such as using remote sensors and combining these with what is known as precision agriculture or satellite farming (Swetha et al. 2020, pp. 62–63). More promising, perhaps, are the wide range of soil test kits that have been developed for on-site low-cost soil testing (Thoumazeau et al. 2019; Columbia University 2020; Smallholder SHA 2021; Vidacycle 2021).

The impact of the current SHC programme is difficult to gauge. Some parts of the country have seen steep declines in fertiliser use and improvements in soil health; others have not. But the scheme’s impact can be measured in other ways. The SHC enhances the capacity of governments to create statistics in a systematic and comprehensive manner and, at least in theory, use them to shape its own authority and the conduct of its citizens. While the state acts at a distance to quantify nature, making new resources available for accumulation or, in the SHC case, intervening in material inputs in response to a fertility crisis, this involves simplifying embedded socio-ecologies, overlooking established soil management practices, and sidelining alternative approaches. Soil testing is more than a technical process: it stakes a claim on who gets to decide what qualifies as legitimate knowledge about the soil (Peña et al. 2017). As the next section explores, the question of how the state is responding to the soil crisis is part of a wider epistemic conflict over who gets to decide what we understand soil to be.

## Environmental governmentality

Responding to the soil crisis requires not just addressing the facts and drivers of environmental destruction. It also requires paying attention to how soil is made known and legible: how soil becomes an object worthy of attention. While there are many mechanisms through which this happens, our focus in this paper is on the role of state and state-related actors. We turn to one of the most powerful conceptual approaches for understanding how the state works in contemporary global environmental politics: governmentality. Deriving from the work of Michel Foucault, the key insight of a now extremely rich body of work is that bodies of knowledge, power and truth inform tactics and practices of governing. Foucault (2007 [1978, p. 144]) suggested that governmentality “has the population as its target, political economy as its major form of knowledge, and apparatuses of security as its essential technical instrument.” For Foucault, the historical constitution of this three-pronged problem in the eighteenth century led to the pre-eminence of ‘government’ as a form of power relation*.* This form of power was exercised through “the ensemble formed by institutions, procedures, analyses and reflections, calculations and tactics” (Foucault 2007, p. 144).

Foucault places the emergence of governmentality in Europe, and the concepts of power he developed can be seen to be specific to that context. However, governmentalities travelled with colonisers and their imposition and translation was partly how colonisation took place (Mitchell 2002). Notions of land and resources and their potential for empire were essential to this process; colonial agrarian production, for example, was transformed by forms of governmentality emerging from those being experimented with in Europe (Agrawal and Sivaramakrishnan 2000). For instance, in his now classic work, Agrawal (2005) documented the emergence of scientific forestry in India. According to Agrawal (2005, p. 43), the “acquisition of knowledge about trees, their occurrence, and their distribution”, was not only critical for the timber industry, but by developing “systematic procedures of identification, enumeration, classification, calculation, and valuation”, statistics could be used to compare the performance of forests and forest departments across India (*ibid*, p. 60). The aim of such quantification was to make exploitable resources legible, and so enhance government’s grasp. More than this, though, Agrawal showed how measuring and classifying forests made Kumaon villagers active partners in protecting their forests, and so led them to become a new kind of environmental subject. Colonial forestry was not simply about quantifying resources, but also about shaping the conduct of those close to those resources.

The homogenising forces of colonial modernity, in the process of meeting the non-Western, were “resisted, reinvented, and reconfigured in different social and historical locations” (Gupta 1998, p. 9). Conventional rule emerged in part as resistance to the way that European models of governance were being imposed in the colonial moment. As Prakash (1999) makes clear, the ‘civilising mission’ of the British was the means through which science took on cultural authority in India. India’s took shape modernity through “railroads, steel plants, mining, irrigation, hydroelectric projects, chemical and petroleum factories, public health organizations and regulations, the bureaucracy and its developmentalist routines, educational and technical institutions, political parties, media and telecommunications, and now, the bomb” (*ibid*., p. 3). On the other hand, religious practices and non-western sciences persisted as alternative sites of authority production, sometimes complementary and sometimes opposed. In this sense it is important to understand India as a “site of scientized religion and a religionized science” (Subramaniam 2019, p. 9). India inherits layered precolonial, colonial, and postcolonial forms of governmentality as well as many local, religious, and other ‘life’ practices (Subramaniam 2019; Legg 2007; Stoler 2002; Gopal 2019).

Governmentality practices in India are thus strongly influenced not only by colonial power relations that embedded specific forms of governing, but other—often anticolonial—modes of rendering bodies productive or collective, including through what Subramaniam (2019) terms bionationalism. This term refers to the processes through which the liveliness of bodies or matter become harnessed to specific state agendas. In India, this has including interpolating nationalism through spiritual practices, such as strict laws about purity and pollution and regimes of body care (*ibid.*). One of the defining features of Hindu nationalism, according to Subramaniam, is the notion of “biology”: within its teachings are important claims about common blood, native ecologies, and unique theories proper to India (*ibid*., p. 10; Münster 2021). Some of these practices became important aspects of resistance to colonial rule, so it is important to understand that there is a clash of claims at the heart of governmentality in India (Prakash 1999). Many alternative spiritual and body-practices, including those that became important in alternative forms of agriculture, emerged partly through this attempt to ground alternatives to colonially-grounded science and the grid it imposed over all forms of life.

As well as a historical phenomenon, governmentality has today obtained a more general meaning—“the ways in which one conducts the conduct of men [*sic*]” (Foucault 2008, p. 186).^1^ The concept has also been widely adapted to understand how nature and environment are governed at a distance through various calculative practices (Dean 2010). These processes can be readily seen in contemporary environmental governance. Environmental problems are often brought to light and made into objects worthy of intervention through statistics and forms of quantitative classification. Forms of knowledge are then used to shape the behaviours and agendas at institutional and individual scales. This occurs across the ‘bewilderingly complex’ suite of today’s environmental problems, from climate change to conservation (Fletcher 2017, p. 314). In the remainder of this section, we set out three axes of how a governmentality approach to environment (environmental governmentality) informs our study of contemporary soil governance in India.^2^

### Knowledge, institutions, expertise and epistemic truth

The first axis of analysis is understanding how the soil is made visible as a problem and environment. Analysis focuses on practical forms of knowledge production, in terms of plans, diagrams, statistical representation, and so on. The key dynamic here is how reality is represented (often by parsing what is included and excluded). Closely related is the need to attend to the wider institutional arrangements, procedures and forms of expertise that inform any given programme of government. Rather than address the global soil crisis in general, an environmental governmentality approach begins from a specific instance of when the question of how to govern the soil is posed. This requires analysis to start from an empirical example, from sites and situations where environmental problems have been “shaped in a thinkable and manageable form” (Rose 1999, p. 22). The point here is not to assess the accuracy of knowledge informing an environmental intervention. It is rather to begin to understand how government programmes, irrespective of their success or failure nevertheless have effects: “they crystallize into institutions, they inform individual behaviour, they act as grids for the perception and evaluation of things” (Foucault 1991, p. 81). Holding these aspects together is the question of epistemic truth: the ‘why’, the goal of a programme, the normative attachment to some forms of knowledge above others, the preferred way of perceiving a problem or environment.

### Subjects and identities

A second key axis of inquiry concerns the kinds of subjects and identities shaped by governmentality. What kinds of subjects are being envisioned? What sort of status, capacity, attributes do people to be governed have currently, and in what ways are these to be changed? Forms of knowledge, institutional power and truth work to interpolate a certain vision of subjects and their conduct; subjects in turn interpolate knowledge, institutional power, and truth. One of the hallmarks of effective governmentality is making subjects behave in a certain way of their own volition, rather than the subjects feeling like they have been made to act by an external force. In other words, successful power is derived by the way that “some actions seem naturally more appropriate than others” (Agrawal 2005, p. 224; Miller and Rose 1990).

### Practice and resistance

The final axis follows from Foucault’s observation that resistance occurs with any exercise of power. Analysis shifts to practice, to places and bodies where messy compromises form and where the logics of governmentality shape life. Resistance is not outside governmentality; it emerges as different practices of self or collective are expanded from existing coordinates. Recent work has focused on how the complexity of subjects and conduct can amplify the possibility for resistance and difference (Larner and Walters 2004). This means understanding the internal aspects of subjectivity, including how social actors perceive their realities and become ethically oriented in relation to them. For example, Agrawal’s classic work on Kumaon forestry has been reassessed to conclude that the government did not successfully shape villagers in the way he originally suggested, in that the villagers were much more reflexive and strategic in their engagement with the forestry programme (Agrawal 2005; see also Cepek 2011). In what follows, the article draws on these three methodological axes (knowledge/institutions/truth; subjects and identities; practice and resistance). It also builds on the recognition that contemporary approaches to governing the soil in India inherit a contested colonial, anti-colonial and postcolonial history.

## Methods

This paper draws on research comprising one component of a larger multidisciplinary project investigating the feasibility of using glacial flour—sediment released into rivers from melting glaciers—as a soil fertility treatment across the Hindu Kush Himalaya.^3^ Other components of the project included glacial flour sample collection, laboratory analysis and field trials. This paper maps the historical, social, and institutional context through which soil health innovations are transmitted in Himachal Pradesh. The state is one of the few in India with a dedicated extension system promoting natural farming within its agriculture department (while simultaneously continuing to promote conventional agriculture). It also has history of using SHCs which predates the nationwide launch by at least seven years. We selected the SHC and ZBNF as two programmes to study because they were the most significant agricultural programmes at state level at the time of our research.

The COVID-19 pandemic meant that we partly adapted our research methods to include Zoom interviews where in person fieldwork was no longer possible. We conducted 38 semi-structured interviews (Table 1). Since we were primarily interested in studying the forms of knowledge, modes of truth production and forms of expertise in soil governance programmes in India, we focused on relevant experts within institutions and networks involved in the development, design, and delivery of soil health programmes.^4^ All interviews were recorded, with consent from the participants, and anonymised if requested. Interviews were carried out in English and fully transcribed. All our interviewees were fluent in English, so although Hindi and other languages are spoken in Himachal Pradesh, no translation was needed. The average length of interview was one hour, broadly structured around three themes: history (historical events, state policies, and actors which have influenced farming practices in relation to soil); practices and knowledge systems (how they think farmers and non-farmers understand and manage soil and soil health, as well as questions about the SHC and ZBNF); and current and future challenges faced by agriculture. Interviewees were heavily weighted towards male (29 male, 9 female) due to the overrepresentation of men in leadership positions and working in agricultural extension. Most interviewees worked either for an environmental NGO, or for the government in the agricultural extension system or within a university. These included interviews with: scientists working for the Natural Farming Unit (*Prakritik Kheti Khushshal Kissan Yojana*) within the Himachal Pradesh Department of Agriculture; soil scientists working for the Krishi Vigyan Kendra (KVK) extension system; scientists working within the Agricultural Technology Management Agency in Shimla; academics at Himachal Pradesh University; and NGOs including the Himdhara Environment and Research Action Collective, and the State Resource Centre. In addition to interviews (Table 2), we draw on a range of secondary sources including government policy documents, political speeches, newspaper articles and websites. Data was initially open coded using Atlas.ti, and we applied thematic and discourse analysis to the interview data and secondary sources. The following sections outline the continuities and discontinuities between soil health approaches.

**Table 1:** List of interviews

Type of interviewee	Number of interviews
Soil/plant scientists	9
Agricultural advisers/extension officers	6
Non-governmental organisations	14
Farmers	4
Politicians/activists	2
Officers in Natural Farming Unit, Himachal Pradesh	3
Total	38

**Table 2:** List of cited interviewees

Interview number	Details
2	Anonymised: farmer and activist who runs a farmer training centre. Interviewed 7 August 2020
16	Anonymised: founder of an Organic Agriculture Research Group. Interviewed 17 August 2020
19	Dr. Prabhakar Rao: Trustee of the SSIAST and Natural Farming Promoter. Interviewed 25 August 2020
22	Dr. K. S. Murali: executive director of M. S. Swaminathan Research Foundation, and Dr. Rengalakshmi Raj, Research Scientist at MSSRF. Interview 13 October 2020
26	Dr. S (anonymised): Researcher within the Agricultural Technology Management Agency and research officer in the Natural Farming Unit. Interviewed 4 November 2020
28	Dr. V (anonymised): Soil Scientist within the Krishi Vigyan Kendra extension system. Interviewed 7 December 2020
29	Dr. K (anonymised): Researcher within the Natural Farming Unit. Interviewed 5 November 2020
30	Dr. C (anonymised): Researcher within the Natural Farming Unit. Interviewed 21 January 2021

Participants have been anonymised where requested

## Epistemics of the Soil Health Card scheme

**Fig. 1:**
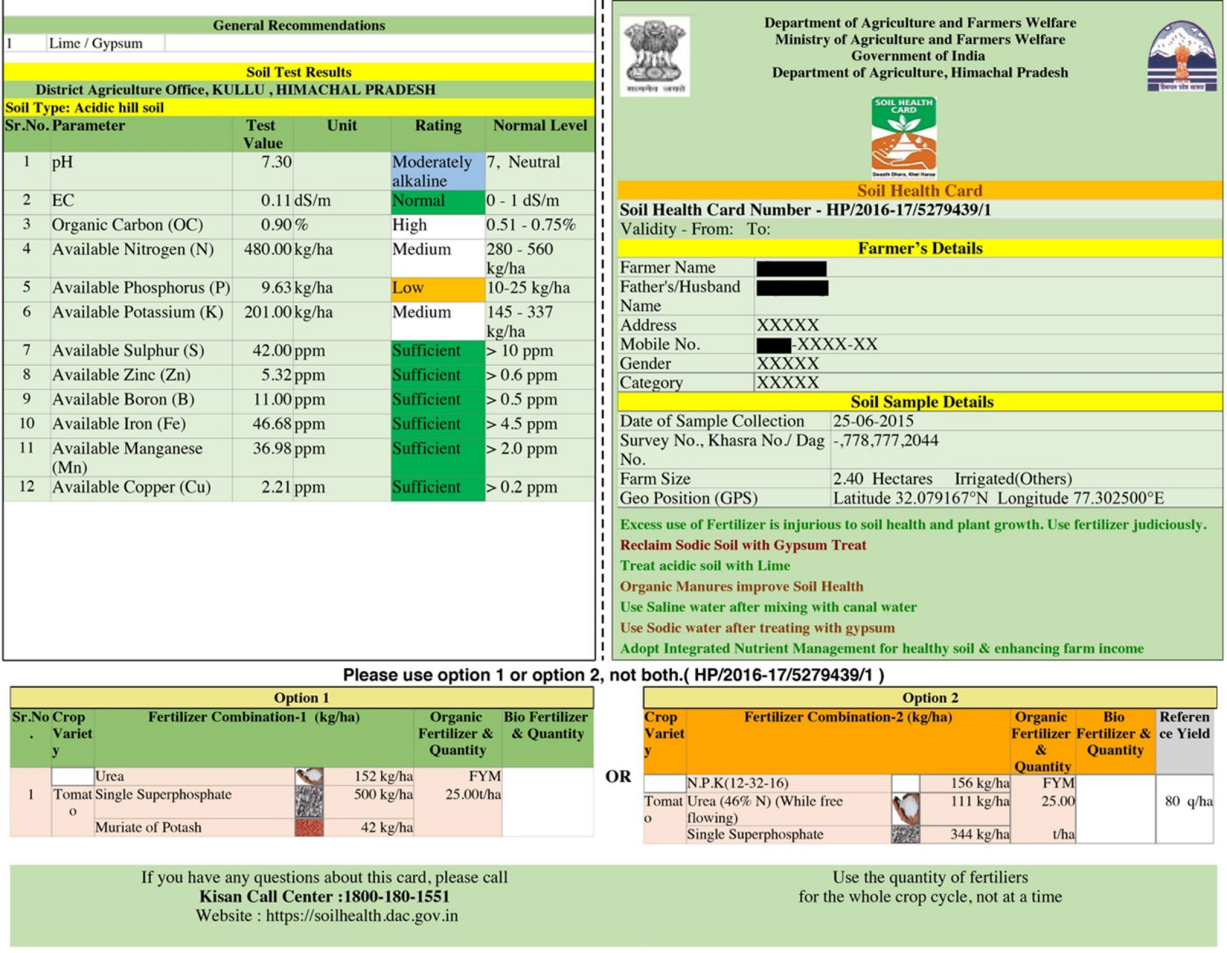
Example of a completed SHC for a farmer in a village in Kullu, Himachal Pradesh. *Source* Soil Health Card ‘print your health card’, https://soilhealth.dac.gov.in/HealthCard/HealthCard/state. Permission to reproduce image obtained

The SHC is a printed report for farmers which provides information on the status of their own soil, based upon samples collected on their farms. The card has details on 12 soil health indicators. These include the three main macronutrients (nitrogen, phosphorous and potassium), the secondary nutrient sulphur, five micronutrients, soil pH, organic carbon, and electrical conductivity. Based on the results of the soil test, the card recommends the types and quantities of fertilisers and soil amendments needed to improve soil health on each farmers’ land. Farmers can access their soil test results through an online database, which allows them to print their results, view fertiliser recommendations, the nutrient status of soil in their village, and find their nearest soil testing laboratory. SHCs are published online, which means that all details of the soil health status of any farmer’s soil (including the farmer’s name) are made available for public viewing—although other identifying details such as addresses and phone numbers are removed (Fig. 1). As an exercise in data collection and management this is a colossal enterprise. According to the scheme’s website, between 2015 and 2020 over 54.5 million samples of soil were tested, with almost 228 million SHCs printed and distributed to farmers (Government of India 2021a, b).

The testing and measurement aspects of the SHC scheme meet two main goals. First, the scheme renders visible areas where farmers can reduce their economic costs by making more efficient decisions about fertilisers (of longstanding concern is overuse of urea as a nitrogen fertiliser). Second, the scheme makes clear where fertilisers are being overused, allowing for more efficient overall production and distribution. Government analysis also linked this inefficiency to the “near absence of soil testing facilities”, and “low awareness” (Ministry of Agriculture and Farmers Welfare 2016, p. 82), making the collection of systematic information the basis for an imagined idea of health. The fertility of Indian soils, which are “showing signs of fatigue,” is understood to be “declining fast” (*ibid.*), which adds urgency to solve the problem. This urgency is supported by evidence linking excessive use of nitrogen fertilisers to diseases and growth impairment in humans and animals.

The scheme renders individual farmers’ practices visible as an area for continuous improvement. The inclusion of farmers’ names places responsibility firmly with individual farmers, and they are envisioned as entrepreneurial, managerial subjects who should make wise decisions about fertilisers. On the other hand, the technical process of administering solutions renders soil health a question of efficiency, input balance and correctly following guidelines, pushing to the side questions of farmer expertise and experimentation. The result is that farmers are ostensibly given more responsibility for their soil while simultaneously being directed about management decisions. This is a classic example of what Stone (2016) labels ‘didactic learning’. This is a process whereby farmer decisions are influenced by parties external to their farming communities. Stone warns that such forms of learning can be maladaptive. Several interviewees who work with farmers in Himachal mentioned negative impacts that can come when government guidance supersedes farmer experimentation (what Stone labels ‘environmental learning’). The founder of an organic agriculture research group recounted how farmers “actually stopped using organic manures, the farmyard manures which they were judiciously and religiously using 50 years ago, because the ICAR [Indian Council of Agricultural Research] forgot to tell them that you use this along with the DAP [Diammonium phosphate] and urea. And the soils have become so sick now” (Interview 16). A farmer and activist who runs a farmer training centre explained how farmers receive subsidised fertilisers based on a single soil sample once they show the results of their SHC but commented that “this is disconnecting farmers from the soil, from the health of the soil even more, because now they’re dependent on somebody to make the test for them and suggest improvements” (Interview 2). This disconnect occurs as soil is quantified and governed from a distance, erasing local, context-specific understanding in favour of a simplified matter of standardisation.

Although the SHC was launched as a nationwide project in 2015, the Himachal Pradesh government had been running a soil testing service since 2011, distributing over 100,000 SHCs to farmers every year. Before that, agricultural extension workers collected soil samples in villages across the state and were distributing cards since at least 2008 (Department of Agriculture 2010). The SHC is generally understood to have been pioneered by the M.S. Swaminathan Research Foundation (MSSRF) in villages around Pondicherry in the southeastern state of Tamil Nadu from the early 2000s. MSSRF’s founder, Professor M.S. Swaminathan, is one of the most influential scientists in India and a “father” of the Green Revolution, serving as the Director of the Indian Agricultural Research Institute from 1961 to 1972, and subsequently occupying key positions within government agencies and ministries responsible for agriculture. Swaminathan explains how “just like our ration card, or our health card, soil should also have the health status card, the farmer should be aware of the status” (Interview 2).^5^ What began as a simple card with only a handful of important indicators, developed into the standardised card that is in widespread use today. Dr. Rengalakshmi Raj, who currently works as a Research Scientist at MSSRF, explained that the organisation expanded on bare-bones soil testing support to include specific advice to farmers about how to improve their soil health based on the results from the tests. Farmers were inscribed as “knowledge workers” within this early scheme, while being trained to monitor the impact of their farming systems on soil fertility with a SHC (Mashelkar 1999, p. 26). Murali, the current executive director of MSSRF, explained that they introduced the SHC as a way of helping farmers “internalise how organic matter plays a major role in facilitating the soil health system”, but that wherever possible they introduce a more microbiological component because otherwise the farmers “would just follow the soil health advisories given by the soil testing laboratory, which is mostly kind of chemical inputs” (Interview 2). This shows that even from its inception, experts were aware of the reductive limits of the SHC and its didactic recommendations. Clearly, through the previous and current incarnations of the SHC the role of the farmer as a manager in a knowledge system was a key concern.

The SHC scheme claims to have increased the efficiency of fertiliser use (Amarender 2017, p. 84; Kumar et al. 2019) and raised awareness among farmers of the need to reduce fertiliser use and increase other micronutrients in soil (Singh et al. 2018, p. 7). However, issues with infrastructure and testing facilities led to delays in returning results from soil testing laboratories, such that farmers did not have the information they needed before the start of the sowing season (*ibid*.; Bera 2015; The Indian Express 2020). This exposes an important contradiction in forms of institutional expertise: the ideal of efficiency in the logic of governing soil is out of sync with the bureaucratic and messy systems of administration through which agriculture is supported in India.^6^ This contradiction is compounded by the fact that the cards have often been distributed with little explanation or training, and that advice does not consider the perspective and knowledge of the farmers (Amarender 2017, p. xv). Indeed, the scheme’s design certainly did not engage the traditional knowhow of farmers.^7^ Secondly, as we will explore, the visibility conferred on nutrient profiles pushes from view other aspects of soil health, such as biodiversity and microbial diversity (not tracked on the card).

This emphasis on the gap between ideals associated with governance programmes and the practices through which they take place reveals something important about soil governmentality. Rather than assessing the extent to which the original blueprint for a programme succeeds in its actual effects, governmentality scholars start with ideas and practices and trace what Foucault called their genealogy, the family history of ideas. These ideas do not necessarily reveal much about what a programme can achieve, but they do reveal the specific logics that are being embedded in particular contexts and that shape how the world is understood. In this case, it is a logic of efficiency as applied to commercially produced nutrients and the notion of farmers as managers of technical solutions. This means that the SHC can be seen as continuing the project of moulding farmers so that their understandings of soil and farming become more and more aligned with the government’s view and with official scientific views. Dr. V, a Soil Scientist within the KVK extension system, explained that in some places the SHC had been beneficial. It had helped to highlight micronutrient deficiencies and make visible aspects of soil health which farmers don’t prioritise. For Dr. V, part of the challenge is that farmers have different priorities: “It takes time, because farmers respond to the effects or whatever they see directly on the plants. They’re more concerned first with disease and pest problems. And whatever they cannot see under the soil *it’s hard to make them understand*” (Interview 28, emphasis added). This view of farmers as recipients of technical expertise marks continuity with the Green Revolution as concerned with modernising and homogenising farming. Initiatives like the SHC also remain aligned with this part of the broader Green—or Evergreen as some prefer to call it (Swaminathan 1996, 2006)—Revolution.^8^

Although the SHC manifests continuity with the colonial-scientific mode of governing agriculture, there are also clear discontinuities: influential framings of the SHC draw on a quasi-religious basis. When Prime Minister Narendra Modi launched the national SHC scheme in February 2015, he referred to soil as both a mother and a human body that needed care. He also equated the SHC to a blood test. Just as modern medical doctors diagnose illness and treatment, Modi said, laboratory soil scientists (“doctors for earth”) give farmers accurate information about the correct dose of medicine (fertiliser) needed to cure the mother/body (soil):

…a farmer should also get his soil tested in the same way, we should check that there is no disease in it, there is no deficiency, there is no problem, and if it is done, then there are doctors for earth who will direct us to do this and that, and it will work for your soil. (Bharatiya Janata Party 2015)

Speaking to an enormous hall full of farmers at a rally in a town in Rajasthan, Modi linked the health of the soil with crop productivity, but also more broadly with the enactment of familial bonds, an indebtedness to the source of life, and the need to care for a sick body:

No matter how much fertilisers we put, how much manure is added … if the earth is not well … then the crop does not grow and there is no production of good crop … This land is my mother. If my old mother is ill at home, I cannot sleep peacefully. If the land is ill, how can I sleep peacefully? That is why, our land, our mother, our soil, it should not be allowed to remain ill … Scientific methods should be adopted to overcome those shortcomings. Like when our body becomes sick … doctors advise to take these medicines and not to take those medicines. As there are rules for the body, likewise there are rules for this mother, for the soil. (Bharatiya Janata Party 2015)

Modi then described the increased income that will be generated from crop yield improvements and more efficient use of fertilisers. While its knowledge and institutional expertise may be modern, these are fused with is a vision of restoring older forms of authority, rendering the nation-state visible as a source of wisdom and good governance, with technicians as medical advisors tending a dear old mother, helping secure Indian soils as the basis for a prosperous future.

This hybrid spiritual-medical analogy shows that while the SHC follows a rational-scientific logic, it can be instrumentalised to strengthen bionationalism—a rendering of nature (bio) that prioritises the protection of the nation-state (nationalism), and a particular set of native or natural practices uncontaminated by global modernity. Leaning on tropes of nature and nativism is a vision one might expect to find in alternative agriculture and soil-care movements, not as part of a major technocratic government testing programme. Invoking bionationalism aligns the SHC with non-mainstream agricultural alternatives in its regime of epistemic truth. It is clear, then, that politicians can multiply the SHC’s forms of epistemic truth and authority, by fusing a regime of empirically-grounded diagnosis with a nativist vision of soil.

## Zero-Budget Natural Farming

The large-scale interventions associated with twentieth century agriculture, and the Green Revolution in particular, were heavily contested. Criticism was accompanied by efforts to preserve traditional practices and experiment with alternative technologies, such as rice intensification systems (Glover 2011; Prasad 2016) and a proliferation of movements expounding ‘natural’ practices as part of an imagined utopian past. From the 1960s, agroecological and natural farming alternatives proliferated in India, in dialogue with other movements around the world. From the 1980s, these networks became increasingly linked through transnational agrarian movements such as La Vía Campesina, which join peasant and indigenous collectives to make shared claims on the global food system (Martínez-Torres and Rosset 2010; Wittman 2009).

The most prominent alternative agriculture network in India is ZBNF, an agroecological movement that invokes a return to agricultural *swaraj* [self-rule] and the revival of explicitly Indian forms of agronomic knowledge and practice as part of the reclaiming of agriculture by and for peasants (Münster 2015, 2021; Khadse et al. 2018). Like La Vía Campesina**,** ZBNF has taken agroecology to scale in India, making it one of the most successful agroecology movements globally (Khadse et al. 2018). ZBNF is akin to a social movement, allowing new associations to pop up quickly (*ibid.*), especially in regions with high rates of farmer suicide, where the emphasis on zero inputs meets the economic concerns of debt-burdened farmers (Münster 2015). ZBNF’s focus on self-reliance (reducing dependence on external inputs, agricultural credit, and diversifying production) emphasises the right of farmers to experiment and come up with solutions—something the Green Revolution eroded. Unlike the epistemics of the SHC scheme, which render soil degradation visible in terms of micronutrients to be calculated and rebalanced, ZBNF highlights the *microbiology* of soil as an overlooked area of activity that has been weakened by successive chemical inputs. Thus, it critiques the lack of attention to soil ‘life’ in national soil health approaches, associating this with a movement of decision-making away from farmers and toward bureaucratic government offices. ZBNF thus repositions soil practitioners (farmers) as experts and experimental scientists as part of its programmes. However, in terms of soil governmentality, the two approaches remain connected in important ways. Critically, they both link soil improvement with embodied spirituality on the one hand, and the heritage of the nation-state on the other. This common element is tied to the history of science and religion in India, and resistance to forms of ordering imposed by colonial elites, as we explain below. As such ZBNF differs considerably from agroecological networks that have emerged in other geographies.

ZBNF philosophy is articulated through the spiritual idiom of its charismatic leader, Subhash Palekar. Palekar leads large training camps that involve long days of instructive talks, rather than the experimental participatory processes more common to agroecology. ZBNF has two principal aims: to maximise farmers’ autonomy from market forces, and to repair soil fertility through cropping design, mulching and the application of the fermented preparation called *jīvāmṛta*, or the “nectar of life” (Münster 2015, p. 243; Bharucha et al. 2020, pp. 5–6). The novel soil care practices of ZBNF reflect an incorporation of microbial health discourses and measurement practices, besides the nutrition elements that the SHC prioritises. Palekar dismisses all soil testing. On his now defunct website he outlined his concerns about the tendency to view soils as lacking in nutrients. He suggested instead that soils lack the micro-organisms to make already existing nutrients available to plants:

Our soil is prosperous - enriched with the nutrients. If the scientific evidences say that the soil is enriched with the nutrients, then why Agriculture University says for soil testing? It is also another fraud. (Palekar 2016)

In focusing on microbial activity as part of the process of diagnosing and remedying soil health issues, Palekar seems to reject bureaucratic forms of knowledge in favour of his own views of soil care.

ZBNF insists on the importance of body purification and soil preparation practices that are linked with mystical higher powers. ZBNF propagates specific forms of nationalism—expressed, for example, in directives to cultivate only truly native varieties of cows, plants, and earthworm (Münster 2021). These can be understood as expressions of bionationalist forms of discipline, which connect particular practices of belonging to a wider body-politic. As such, when farmers are convinced by the effectiveness of *jīvāmṛta* on their soils, they may also buy into new diets, or even ideological principles “compatible with nativist politics and the tropes of the Hindu Right” (Münster 2021, p. S311). Münster (2018) goes so far as to say that ZBNF involves the development of a new agroenvironmental subjectivity tied to distinct spiritual commitments. He argues that Palekar camps function like ‘revival meetings’ where farmers are “repeatedly invited to stand up, raise their right arm and solemnly vow to transform themselves from being a ‘demon destroyer of nature’ to a ‘saint protector of nature’” (Münster 2018, p. 755). This commitment to moral protection restricts materials that can be included in agriculture. ZBNF rejects the authority of research institutions, the extension system, agricultural development agencies and the private sector, emphasising instead microbial health and guru-led spiritual practices. Of course, even as this alternative form of agriculture encourages soil health and farmer empowerment, it may also be producing its own forms of exclusion and control. After all, ZBNF has its own fairly rigid prescriptions for the correct way of carrying out natural farming, from the recipe for *jīvāmṛta* and instructions on its use, to the correct varieties of earthworks and cows—the “demonic and abominable non-Indian, nonnatural, no-cow species” (Münster 2017, p. 30).

Although associated with Subhash Palekar, the core principles of zero-budget natural farming have been adopted by a wide range of organisations, and many do not take up the spiritual language with which Palekar imbues his teachings. Some, like the not-for-profit company Rythu Sadhikara Samstha run by the government of Andhra Pradesh, have adapted it slightly to emphasise the climate resilient aspect of ZBNF (APZBNF 2020). Others have their own philosophies; the Art of Living Foundation, for example, promotes a form of natural farming it calls Sri Sri Natural Farming (SSIAST 2021b), which works with over 2.2 million farmers (SSIAST 2021a). Dr. Prabhakar Rao, a trustee of the SSIAST and natural farming promoter, eschews phrases such as ‘spiritual power’ or ‘message from God’. Rather, natural farming is framed as both ancient—linking it to Vedic agriculture—and scientific, with talk of experiments and statistical analysis (Interview 19). Nevertheless, Rao claims that whatever their differences, natural farming approaches provide an alternative to a paradigm that makes soil health dependent on the application of chemical fertilisers which denude the soil of critical ‘nutrient solubilizing microbes.’ In his view, farmers should avoid using the SHC entirely:

and just tear up that Soil Health Card because that has got no relevance to the technology we’re using, because our job is to put in the microbes, let the microbes do the work, and the microbes will extract every single nutrient from the soil. It’s not that we need to have a chart that tells us you know what to do … if you’re using a technology that is basing itself on the microbes … then the SHC is not even relevant. (Interview 19)

The statement suggests that the way the SHC makes the soil visible as a ‘problem’ is being rejected by many farmers. They refuse to be governed at a distance by a reductive representation of soil health, and instead prioritise empirical experience and monitoring of the material agency of soil microbes.

On the other hand, in other ways, ZBNF reproduces the governmental logic in play in the SHC, especially in terms of the way the nation-state is framed. Like the bionationalist epistemics we traced in the SHC in the previous section, ZBNF is spoken about as a way to revitalise a tired and bureaucratic *nation*, and as in the SHC this is also linked with body purification practices, associated with Indian spirituality. Since 2014, ZBNF has even been incorporated by many regional states to meet the growing challenge of soil degradation. In Himachal Pradesh, ZBNF was rebranded *Prakartik Kheti-Khushal Kisan Yojna* (Natural Farming Happy Farmer Programme), within the Government’s Agriculture Department in 2018 (Government of Himachal Pradesh 2018a). Himachal Pradesh became the first state to promote natural farming directly through its Natural Farming Unit, rather than via a non-profit organisation, via a dedicated unit within the Directorate of Agriculture (Government of Himachal Pradesh 2018b, p. 4). This was met with rapid uptake: over 100,000 rural farmers now practising natural farming on around 5400 ha of land (Interview 30), equivalent to around 10% of all farmers on 0.6% of the agricultural land of the state (Department of Economics and Statistics 2018).

As part of this restructuring, NGOs and Farmers Societies working on organic or natural farming were reimagined as part of a strategic renewal of state plans, especially by providing training—for example, some 284 extension officers were trained in the practices of natural farming. Despite being situated as a counter-position to the SHC, ZBNF is increasingly incorporated into the network of state institutions: the ‘resistance’ it offers is an extension to the existing logic of governmentality, not a break with it. Universities were also contracted in this process to provide trials of ‘location specific technology’ and recommend ‘package[s] of practices’ for different crops (*ibid.*, p. 7). Agroecology is increasingly portrayed as a way to make local agriculture more *productive*—an axis of success in the SHC: to launch a ‘sustainable food system platform’ that would assist farmers market their produce, the state provided farmers with a wide range of incentives such as infrastructure and inputs for bio-fertilisers and bio-pesticides (*ibid.*, p. 7). Dr. C observed this transformation, noting that, rather than subscribing to the forms of spiritual or politicised version of ZBNF, in practice farmers assessed their programmes according to their productivity: “If a farmer finds it’s better, if farmer finds a good consumer, if farmer finds that he is getting more income by doing this, automatically, he will shift [his practice]” (Interview 30).

Indeed, part of the success of ZBNF in Himachal Pradesh can even be attributed to the way the programme piggybacked on the state’s existing administrative structures. Thus, the new programme is being developed *alongside* long-standing policies promoting conventional agricultural ideas such as a ‘package of practice’ for each crop, Green Revolution type technological interventions, and the SHC—which have not been radically displaced by its success. This means that multiple, concurrent and sometimes competing forms of authority are in play structuring the opportunities and governing logics in agriculture. Thus, although scientists like, Dr. C, a an entomologist and pesticides expert working within Himachal Pradesh’s Natural Farming Unit, emphasised that the SHC and natural farming are in many ways non-compatible, we increasingly see farmers using elements of both programmes, in a ‘mix-and-match’ style. Although both programmes—in principle—aim to reduce the use of chemical fertilisers, in practice what is happening is the new visibility of rural farmers encourages a disposition to experiment. Agroecology is incorporated into a nationalistic programme where bureaucracies still thrive—but farmers feel increasingly empowered to make their own choices, based on embodied assessments of soil texture and plant growth.

This incorporation is not consistent, and in some areas the truth regime of ZBNF is more explicitly associated with disagreement and autonomous practice. Dr. S, who works for the Agriculture Technology Management Agency and carries out extension work for the Natural Farming Unit, considers the institutional technics of ZBNF as totally distinct from those of the SHC. He emphasised that soil testing is done by a separate department, and that in their system (natural farming), people have refused to comply with SHC requirements:

**Author 1**: I’m interested because soil testing now is a big programme but it isn’t part of the natural farming programme. I’m interested in how the two work together.

**Dr. S**: In our system, people they stopped giving their soil sample…

**Author 1**: The farmers that are doing the natural farming don’t need to do the soil testing anymore?

**Dr. S**: No, no. They are not interested in it.

**Author 1**: Because this soil testing would say you have to put this much nitrogen...

**Dr. S**: Yes, nitrogen, potassium, phosphorous...and all that. They stopped all that (Interview 26).

Actors like Dr. S. emphasise that the relationship of care and attention that farmers are encouraged to develop with their soils in ZBNF is distinct to the processes of technical testing and mineral supplement prescribed by the SHC. Within the SHC soil testing laboratories, report cards and application recommendations render soil visible as a material substrate to be improved via the addition of external agents. The SHC, with the technical support of scientists and extension officers, becomes both a repository and the ultimate arbiter of soil knowledge, stewarding farmers towards a relationship of soil care in line with established scientific practice.

There is, thus, some question about how far the two programmes can and do co-exist. They are based on incompatible forms of knowledge about the soil, and they compete for legitimacy through farmer’s endorsement. However, despite these contradictions, both continue to run in parallel in states like Himachal Pradesh and farmers are able to pick and choose. Another scientist within the Natural Farming Unit, Dr. K, concluded that it was likely that distinct systems of agriculture—chemical-based, organic, and natural farming—were likely to persist alongside one another because it is up to individual farmers and whether they “feel good” about the programme in question (Interview 29). He continued, “The department is currently giving support to all types of farmer. The department cannot force. In Natural Farming unit, we are promoting our technology. Farmers are coming and adopting our technology in large number… We will make them aware about our technology. The decision lies with the farmer. This point lies at the core of the fracturing of expertise we observe; the multiplication of different forms of epistemic truth in soil care makes it impossible to discern a single ‘solution’ to soil crisis, for the forms of knowledge posit the ‘problem’ of soil health so differently. However, it is critical to note that the evolving governmentality places new emphasis on rural farmers’ decision-making, and on equipping them to assess the options available. This appears to be a contribution of ZBNF and related programmes, which can be seen to rework the logic of earlier programmes, creating new subject positions, without truly disrupting their forms of visibility or institutional networks of expertise. The form of resistance offered by ZBNF does not, in this case, extend beyond dominant logics of governing.

It is not insignificant, however, that the rural farmer is conceived and shaped as a subject very differently through ZBNF, despite its relative institutionalisation. The SHC programme conceives of farmers as managers that respond to rational, quantified directions by implementing technical decisions. In natural farming programmes, by contrast, the farmer is exposed to various possible farming solutions, compares these new ideas in relation to existing practices and then decides on the best solution for their farm. This also means that farmers are enabled and legitimised to decide how the soil is known rather than being required to accept external regimes of knowledge, in turn fostering a proliferation of programmes that are construed as options, rather than a direct competition between opposing programmes. There remain additional differences *between* natural farming approaches: guru-oriented approaches like ZBNF focus on large camps and the charismatic teaching of Subhash Palekar, whereas the Natural Farming Unit has adapted to an existing technical extension system using a flexible strategy, retaining more autonomy for the individual farmer. This autonomy is an important feature of agroecological networks and transnational agrarian movements around the world: rather than simply repair soil ecologies degraded by extractivist models of production, the idea is to initiate a knowledge revolution that takes back the right to choose what and how to grow (Millner 2017). Yet in India, and in Himachal Pradesh as this article has shown, this seems to be fostering a new ‘market of ideas’ rather than a distinct political movement that is demanding or claiming autonomy in any substantive sense.

## Conclusion

In this paper we have followed two soil remediation programmes in Himachal Pradesh, India, to explore their power-knowledge relations and types of subject formation. We explored how the SHC reproduces elements of colonial scientific administration, positioning farmers as managers but not experts, in a system that frames soil as a substrate to be improved through the addition of nutrients depleted via over-fertilisation and continuous cropping. This form of governing soil health seems at first directly at odds with the forms of natural farming also being implemented by states such as Himachal Pradesh, leading to contradictions in agricultural practice and forcing farmers to choose between different approaches. However, through our analysis we have shown that natural farming, especially as embodied in state programmes like Himachal Pradesh’s Natural Farming Unit, shares important parallels with the SHC (and with conventional or Green Revolution style agricultural thinking).

The SHC sees farmers as functionaries and managerial subjects and soil as a substrate for production—composed of quantified inputs and processes, but nothing more. The SHC is a programme to repair the fertility of soil and address the soil crisis which is rooted in colonial-era technocratic programmes, and sidelines questions of farmer experimentation, expertise, and the diverse histories of soils. The SHC is, ultimately, rooted in the same extractive, productivist logics as conventional agriculture. ZBNF, by contrast, emerges from alternative movements experimentally developing in India. ZBNF, as promoted through grassroots peasant organisations, looks beyond issues of soil health alone to include food sovereignty, the rights of farmers, climate change and biodiversity conservation. The micro-practices of movements like ZBNF reveal dynamic interactions with the microbial matter of soils, reflecting cutting-edge ecological science, and developing modes of soil repair that are more long-term than those that prioritise replacing lost nutrients with chemical fertilisers. However, while farmer agency and the soil itself is given more due, ZBNF does not escape the logic of a managerial approach: its radical aims are being taken up by state governments which attempt to accommodate alternative soil regimes with mainstream soil science, and many of its prescriptions are rather didactic, from the recipes for bio-inputs to the species of cattle and worm considered acceptable (Kearnes and Rickards 2020; Brown 2018).

Despite their differences, both the SHC and ZBNF share an appeal to spiritual values imbued in soil and country specific to the Indian context. They both ground authority in what we term in this paper—after Subramaniam (2019, p. 10)—bionationalism, a concept denoting the deployment of nativist views of life with nationalist rhetoric. Bionationalist practices connect the moral imperative of soil repair with ideas of the ‘mother’ earth and other spiritual practices that designate native varieties of animal or seed as desirable. This means that the seemingly alternative practice of ZBNF reinforces forms of nationalism that may be exclusionary or tend toward associating good agriculture with specific ideological and moral agendas. Moreover, while the two approaches may appear contrasting, in practice they are overlapping options to be accommodated (or not) within farming practice. Noting these parallels helps us observe the emergence of governmentality that illuminates certain figures as experts. State and science make the soil visible and embody legitimate forms of expertise in the SHC. State-led ZBNF, by contrast, trains farmers to understand themselves as innovators, learning to experiment and adapt solutions to fit their own specific needs and environments. In this sense a contradiction opens between the two programmes around the question of expertise: where the SHC emphasises the farmer as an implementer of solutions designed elsewhere, the natural farming focus on in situ experimentation reframes the farmer as a decision-maker. However, this contradiction has been rendered into sets of options to be considered, rather than political positions that are opposing in any meaningful sense. Thus, in states like Himachal Pradesh, where both programmes are in operation, we witness a shift toward hybrid formats and pick-and-mix approaches, as farmers and their organisers are increasingly invested with the capacities to choose and combine approaches. We see, then, a fracturing of expertise and the opening up of epistemic pluralism in the response to soil fertility crisis.

## Abbreviations

KVK: *Krishi Vigyan Kendra* (Agricultural Extension Center)
SHC: Soil Health Card
SSIAST: Sri Sri Institute of Agricultural Sciences and Technology Trust
ZBNF: Zero Budget Natural Farming

## Footnotes

1 A key moment in the history of this approach was the (1991) publication of *The Foucault Effect: Studies in Governmentality*, edited by Burchell et al., which followed Gordon’s detailed synthesis of Foucault’s writings between 1972 and 1977 (Foucault, 1980). Key interventions from Rose (1990) and Dean (2010 [1999]) paved the way for contemporary studies of diverse phenomena using a governmentality approach (see Larner and Walters 2004 on the development of governmentality studies across the disciplines).

2 The contours of the debate between environmentality, environmental governmentality, green governmentality and others do not detain us here. See Fletcher (2017) for an overview.

3 The ‘Glacial Flour Power’ project was a transdisciplinary investigation of the potential of glacial flour in supporting crop yields and promoting soil care in the Hindu Kush Himalaya (2019–2021).

4 We had initially planned a secondary focus on the experience of farmers in the same regions, but the rising COVID-19 pandemic made this practicably impossible. We were able to gain mainly meaningful insights into rural experience via our organisational interviews but this aspect of our initial methodology could not take place within the project timeline.

5 As chairman of the National Commission on Farmers from 2004 to 2014, Swaminathan called for the Government of India to set up a “national network of advanced soil testing laboratories with facilities for detection of micronutrient deficiencies” among many other recommendations (PRS Legislative Research, 2006, p. 2).

6 A recent report lists the problems: shortage of labour for collecting soil samples; shortage of resources for soil testing (infrastructure, machinery, labour); poor coordination between extension officers and testing labs; over-emphasis on distribution targets rather than explaining the content to farmers; poor understanding of the cards by farmers; low farmer confidence in using the cards; an overburdened extension system; and low levels of trust in the recommendations returned from soil sample (Nair et al. 2019).

7 Despite the volume of academic papers and research on soil management and soil-related topics, there are few papers on the indigenous or traditional knowledge of soil, or ethnopedology, of farmers in India (Dey and Sarkar 2011; Kuldip et al. 2011; Nath et al. 2015).

8 In 1996, Swaminathan coined the term ‘evergreen revolution’ (Subramaniam 2019, p. 53) to emphasise the need to develop technologies that could “achieve productivity in perpetuity without ecological and social harm” (Swaminathan 2006, p. 2293).
